# Reduction in Hospital-Wide Clinical Laboratory Specimen Identification Errors following Process Interventions: A 10-Year Retrospective Observational Study

**DOI:** 10.1371/journal.pone.0160821

**Published:** 2016-08-05

**Authors:** Hsiao-Chen Ning, Chia-Ni Lin, Daniel Tsun-Yee Chiu, Yung-Ta Chang, Chiao-Ni Wen, Shu-Yu Peng, Tsung-Lan Chu, Hsin-Ming Yu, Tsu-Lan Wu

**Affiliations:** 1 Department of Laboratory Medicine, Linkou Chang Gung Memorial Hospital, Taoyuan, Taiwan; 2 Department of Medical Biotechnology and Laboratory Science, Chang Gung University, Taoyuan, Taiwan; 3 Department of Nursing, Linkou Chang Gung Memorial Hospital, Taoyuan, Taiwan; 4 Department of Nursing, Chang Gung University of Science and Technology, Taoyuan, Taiwan; 5 Department of Management Information System, Chang Gung Medical Foundation, Taipei, Taiwan; 6 Department of Medical Laboratories Administrative Center, Chang Gung Medical Foundation, Taipei, Taiwan; University of Manchester, UNITED KINGDOM

## Abstract

**Background:**

Accurate patient identification and specimen labeling at the time of collection are crucial steps in the prevention of medical errors, thereby improving patient safety.

**Methods:**

All patient specimen identification errors that occurred in the outpatient department (OPD), emergency department (ED), and inpatient department (IPD) of a 3,800-bed academic medical center in Taiwan were documented and analyzed retrospectively from 2005 to 2014. To reduce such errors, the following series of strategies were implemented: a restrictive specimen acceptance policy for the ED and IPD in 2006; a computer-assisted barcode positive patient identification system for the ED and IPD in 2007 and 2010, and automated sample labeling combined with electronic identification systems introduced to the OPD in 2009.

**Results:**

Of the 2000345 specimens collected in 2005, 1023 (0.0511%) were identified as having patient identification errors, compared with 58 errors (0.0015%) among 3761238 specimens collected in 2014, after serial interventions; this represents a 97% relative reduction. The total number (rate) of institutional identification errors contributed from the ED, IPD, and OPD over a 10-year period were 423 (0.1058%), 556 (0.0587%), and 44 (0.0067%) errors before the interventions, and 3 (0.0007%), 52 (0.0045%) and 3 (0.0001%) after interventions, representing relative 99%, 92% and 98% reductions, respectively.

**Conclusions:**

Accurate patient identification is a challenge of patient safety in different health settings. The data collected in our study indicate that a restrictive specimen acceptance policy, computer-generated positive identification systems, and interdisciplinary cooperation can significantly reduce patient identification errors.

## Introduction

There has been increased interest in issues involved with reducing patient errors and improving patient safety since publication of a report entitled “To Err is Human: Building a Safer Health System” by the Institute of Medicine [1]. Patient identification (ID) and accurate specimen labeling during phlebotomy procedures are crucial first steps in the prevention of medical errors [2]. At least two patient identifiers should be used before collecting a specimen [3, 4]. All identifying labels must be attached to specimen containers at the time of collection rather than at a later time [5, 6]. ID errors related to laboratory specimens may involve misidentification of a patient or a patient specimen. Patient ID errors and sample mislabeling at the time of collection remain a serious problem in most clinical laboratories. A lack of standardized definitions and systems to detect or report ID errors lead to great variability in ID procedures and in published ID error rates among institutions and service settings [7–10]. Patient and specimen ID errors have been reported at rates of 0.005–1.12% among various institutions, and many more such errors may be underreported [11–14].

Misidentified specimens not only adversely impact patient care but also increase the cost of health care delivery. ID errors can have serious consequences for patients, including missed or delayed diagnosis; incorrect or unnecessary treatment; patient injury; and severe transfusion reactions [9, 11, 13–17]. In a large multicenter study, 55.5% of ID errors were reported to be associated with primary specimen labeling errors. Moreover, it is estimated that approximately 1 in 18 ID errors result in adverse events [11] and the cost of misidentified specimens is estimated to be around 280000 USD per million specimens [18].

Most ID errors occur in the wards or the emergency department (ED)[19, 20]. These departments are labor intensive and not under the control of the clinical laboratory. Reducing patient misidentification could be approached using non-technical methods (patient safety guidelines and procedures) and/or technical solutions (ID wristbands containing a barcode), as suggested by Da Rin [21]. In a 2012 systemic review and meta-analysis of laboratory medicine best practices, it was concluded that barcoding is effective for reducing patient ID errors in diverse hospital settings [22].

Only a few published studies have focused on approaches to minimizing patient ID errors during specimen collection in different health care settings. The current study was implemented at Linkou Chang Gung Memorial Hospital (CGMH), including the outpatient department (OPD), inpatient department (IPD) and emergency department (ED). Patient ID errors occurred with approximately 100 samples each month in 2004. For advancing standards of patient care, the head of laboratory medicine at CGMH proposed the initiation of a series of interventions to be carried out together with the departments of nursing and management information systems. We hypothesized that the introduction of improved methods for accurate labeling in the hospital would significantly reduce patient ID errors. It is important to share experiences of reducing ID errors from different health organizations worldwide, to identify the most culturally suitable procedures for minimizing laboratory and patient ID errors.

## Materials and Methods

### Study Sites

The current study was conducted at CGMH, a 3800 bed academic medical center in Taoyuan, Taiwan. Electronic computer physician order entry (CPOE) has been in place since 1998 throughout the entire hospital. During the study period, about 2500 patients a day were attended by well-trained phlebotomists in the outpatient phlebotomy services unit within the Department of Laboratory Medicine. Inpatient phlebotomy services in the ED and wards were provided by nurses.

This study included patient specimens from the OPD, ED and IPD that were received by the Department of Laboratory Medicine from January 2005 through December 2014. These included all specimens for chemistry, hematology, coagulation, immunology, virology, microbiology, molecular, and STAT testing, as well as urinalysis, toxicology, and blood bank specimens. There were 3761238 specimens recorded by the laboratory accession system in 2014.

### Data Collection

Since 1 January 2004, the Department of Laboratory Medicine as maintained extensive quality assurance (QA) and quality control (QC) records documenting all patient specimen identification errors according to its incident reporting system, including those that occurred in the OPD, ED and IPD during the study period. Documentation of all laboratory requests received, sample rejections, specimen labeling errors, and test cancellation rates from the entire hospital are available in Department of Laboratory Medicine QA/QC monthly reports. This study was approved by the Ethics Committee of Chang Gung Memorial Hospital (IRB no. 104-7156B). The IRB waive the requirement to obtain the signed consent form because the data were analyzed anonymously.

### Definition of patient ID errors

There are three common categories of patient ID errors: (i) mismatch between the requisition and the specimen label; (ii) unlabeled specimens; (iii) mislabeled specimens or wrong blood in tube (WBIT), meaning patient A’s label is on the tube with patient A’s requisition but patient B’s blood (specimen) is in the tube [23].

### Intervention I (April 2006): restrictive specimen acceptance policy in the ED and IPD

We searched the literature and found that it has been proved that a restrictive specimen acceptance policy can reduce the specimen ID error rate [24, 25]. A restrictive specimen acceptance policy agreed on by directors of both the nursing and laboratory medicine departments was implemented on 1 April 2006. Relabeling of mislabeled or unlabeled specimens was not allowed, except for in cases of irretrievable specimens (e.g., cerebrospinal fluid, tissue, or blood cultures taken prior to antibiotic therapy). If relabeling was necessary for these specimens, the policy required health care personnel to come in person to the laboratory to identify the specimen and to sign the incidence record. All ID errors documented by health care personnel were reported to their own departments.

### Intervention II (August 2007): computer-assisted barcode positive patient ID system in the ED

In 2007, the goal of CGMH was to create a paperless hospital. From previous studies, we learned the advantage of combining the positive patient identification system and computer-assisted bar-coding system [26, 27]. Implementation of the computer-assisted barcode positive patient ID system was initiated in the ED on 1 August 2007, with software developed by the hospital information system (HIS) staff. We combined the CPOE system with the HIS to produce barcoded ID wristbands, which were printed at the start of each patient ED visit. Physicians ordered tests using the HIS. Then nurses could read the order and prepare specimen barcode labels with a nursing cart, rather than completing requisition forms at the ED nursing station. The specimen label included the patient’s name, medical record number, requisition number, type of specimen required, test request, and a lab barcode. Patient verbal ID was manually compared with the patient wristband and sample label information while labeling of collection tubes took place at the bedside. The nurse would use a wireless barcode reader scan their own employee ID badge, then scan the patient wristband barcode, and lastly scan the barcode label on collection tubes to identify the patient. If the ID did not match that of the patient, a warning alarm and message would be displayed on the nursing cart screen. Once the ID is matched, the nurse could proceed to collect the samples, and the time of barcode scanning was recorded to the laboratory information system (LIS) as the specimen collection time. The specimen barcodes were then scanned again before being delivered to the laboratory by a pneumatic tube system and the time was recorded in the LIS for sample tracking. Laboratory staff received and handled specimens by directly scanning each label.

### Intervention III (June 2009): automated sample labeling combined with electronic identification system in the OPD

The OPD phlebotomy station was equipped with a specimen barcode printing instrument connected to the CPOE system on 27 January 2004. The phlebotomist printed a barcode and placed barcode stickers on the collecting tubes. Two automated tube selecting and label tracking systems (BC-ROBO®787, Techno Medica, Yokohama, Japan) were introduced at the OPD phlebotomy station, replacing manual barcode labeling in June 2009. The BC-ROBO®787 tracking system automatically prepared and transported a complete “patient kit”, a plastic box containing a complete set of barcode-labeled tubes along with a work list based on the physician’s test order. Patient ID was not only via verbal positive ID but an electronic ID system was also used. At the OPD phlebotomy station, patients did not wear an ID wristband; they must carry their own national health insurance card. An electronic ID system in the OPD, which was developed by hospital information staff, read the patient’s national health insurance card information and then scanned the barcodes on work list from the patient kit. If the ID did not match the patient, error message “X” would be triggered on the screen at the phlebotomist’s desk. If the ID matched the patient, a correct message “O” would be displayed.

### Intervention IV (September 2010): computer-assisted barcode positive specimen ID system at inpatient nursing stations

Because intervention II was so successful in the ED, the barcode system was implemented at inpatient nursing stations in September 2010. It took 1 year to complete the system at approximately 300 nursing stations. The intervention process on the wards was similar to that in the ED except that paper requisitions printed by the CPOE were still required. This system was not used in intensive care units (ICU) because ICU operational systems are completely different to those of other wards.

### Statistical Analysis

We performed statistical analysis with Microsoft EXCEL and SAS software version 9.2 (SAS Institute, Cary, NC, USA). For descriptive statistics of variables, results are reported as numbers and percentage. The reduction (%) of errors was calculated by the equation ([pre-intervention error rate–post intervention error rate] / pre-intervention error rate × 100). We applied the two-proportion z test for the difference between two proportions as probability tests of errors between two different periods. In all analyses, a two-tailed *P* value <0.05 was considered statistically significant.

## Results

### Identification error rates at different service sites

In 2005, over 2 million clinical laboratory samples were collected at CGMH during the pre-intervention period. Of the total samples, 1023 (0.0511%) were reported as having patient ID errors, an average of 85 samples a month. Although the ED provided only 20% of the specimens, it had 41% of patient ID errors during 2005 (an average of 35 samples a month). Most of the total samples (47%) came from the IPD, which was also the department that accounted for 55% of those samples with hospital patient ID errors during 2005 (Table 1). Samples from the OPD accounted for 33% of the total, but ID errors in the OPD accounted for only 4% of all errors in 2005 (Table 1).

**Table 1:** Comparing patient identification errors between 2005 and 2014.

Sites	January-December, 2005			January–December, 2014			Reduction	P
	Errors	Specimens	Error%	Errors	Specimens	Error%		
**ED**	423	399636	0.1058	3	444017	0.0007	99%	<0.001
**IPD**	556	947156	0.0587	52	1158402	0.0045	92%	<0.001
**OPD**	44	653553	0.0067	3	2158819	0.0001	98%	<0.001
**Institution**	1023	2000345	0.0511	58	3761238	0.0015	97%	<0.001

Abbreviations: ED, emergency department; IPD, inpatient department; OPD, outpatient department.

### Serial interventions to minimize hospital-wide patient ID errors over 10 years

**Fig 1:**
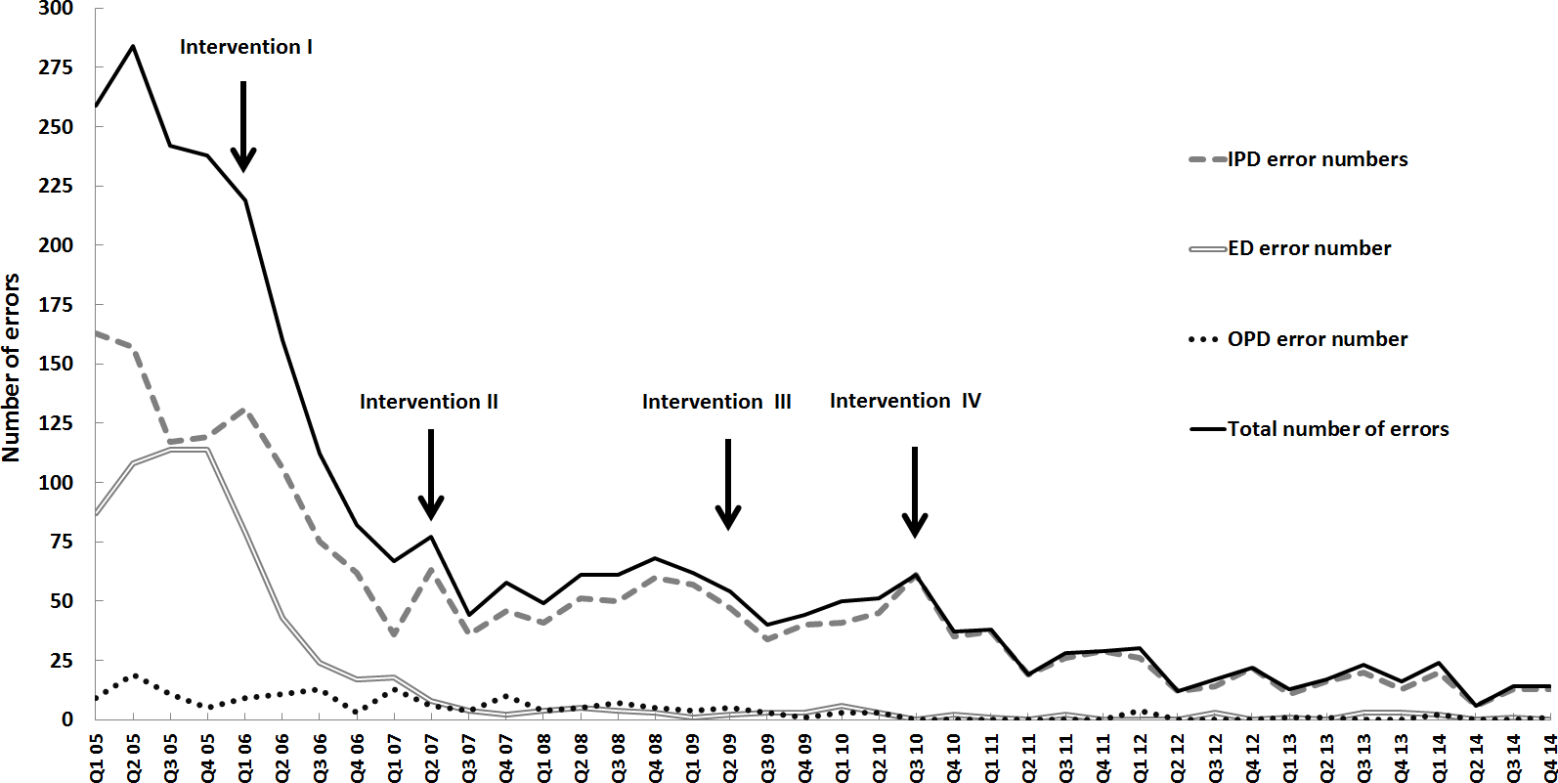
Quarterly errors (*y*-axis) in patient identification over a 10-year period at Linkou Chang Gung Memorial Hospital. Arrows indicate the start of the four interventions.

After comparison of the serial interventions implemented from 2005 to 2014, the total numbers and rates of hospital patient identification errors had dramatically decreased from 1023 to 58 and from 0.0511% to 0.0015%, respectively. The total number (rate) of patient identification errors in the ED, IPD, and OPD before and after interventions over the 10-year period were 423 (0.1058%), 556 (0.0587%), and 44 (0.0067%) compared with 3 (0.0007%), 52 (0.0045%), and 3 (0.0001%) respectively, representing a relative error reduction of 99%, 92% and 98%, respectively (Table 1). Fig 1 displays quarterly data of patient ID errors over the 10 years, and shows a drastic reduction in errors after intervention I (restrictive specimen acceptance policy) in the ED and IPD, proving the success of this intervention to be sustainable. Intervention II in the ED and intervention III in the OPD, both positive identification systems, had successfully decreased ID errors in the ED from 18 in the first quarter to 2 in the fourth quarter of 2007; in the OPD, errors decreased from 5 in the second quarter to 1 in the fourth quarter of 2009. After intervention IV, there was a steady decrease in patient specimen ID errors that was coincident with introduction and slow familiarization with the new positive ID barcode labeling system on the wards. In 2014, there were only 3 specimen mislabeling errors in both the ED and OPD. In contrast, the ICU had not implemented the barcode ID system so the total number of errors (52) and error rate (0.0045%) in the IPD was higher than those in the OPD and ED (Table 1).

### Reduction of patient ID errors by intervention I

To further analyze the effects of the interventions on reduction of specimen ID errors, baseline data were collected for a 1-year period (April 2005 through March 2006 as the pre-intervention I period and April 2006 through March 2007 as the initial post intervention comparison period). After intervention I, the total number (rate) of mislabeled specimens in the ED and IPD had significant decreased from 415 (0.1034%), 524 (0.0546%) to 102 (0.0246%), 279 (0.0280%), representing a relative reduction of 76%, and 49%, respectively, with a significant difference in the proportion of errors for the two periods (P <0.001; Table 2).

**Table 2:** Specimen identification errors before and after serial interventions.

Intervention	Period	Sites	Errors (%)	Specimens	Reduction (%)	*P*
Pre-intervention I	Apr. 2005–Mar. 2006	ED	415 (0.1034)	401252		
Post-Intervention I	Apr. 2006–Mar. 2007	ED	102 (0.0246)	414162	76%	<0.001
Pre-intervention I	Apr. 2005–Mar. 2006	IPD	524 (0.0546)	959930		
Post-Intervention I	Apr. 2006–Mar. 2007	IPD	279 (0.0280)	994901	49%	<0.001
Pre-intervention II	Aug. 2006–Jul. 2007	ED	64 (0.0151)	424738		
Post-Intervention II	Aug. 2007–Jul. 2008	ED	14 (0.0033)	419905	78%	<0.001
Pre-intervention III	Jun. 2008–May. 2009	OPD	18 (0.0012)	1501198		
Post-Intervention III	Jun. 2009–May. 2010	OPD	11 (0.0007)	1601059	43%	0.141
Pre-intervention IV	Sep. 2009–Aug. 2010	IPD	185 (0.0164)	1125755		
Post-Intervention IV	Sep. 2010–Aug. 2011	IPD	127 (0.0112)	1135092	32%	<0.001

Abbreviations: ED, emergency department; IPD, inpatient department; OPD, outpatient department.

### Reduction of patient specimen ID errors by intervention II

The barcode patient specimen ID system was first implemented in the ED during August 2007. We compared the data collected in the 1-year period prior to implementation (August 2006 to July 2007) and post implementation (August 2007 to July 2008) in that department. The total number (rate) of ID errors in the ED decreased from 64 (0.0151%) to 14 (0.0033%), with a relative reduction of 78% and a significant difference in the proportion of errors for the two periods (P <0.001; Table 2).

### Reduction of patient specimen ID errors by intervention III

At the OPD phlebotomy station, we introduced the integrated national health insurance card and automated sample labeling system to replace barcoded ID wristbands for patient identification. Total baseline ID errors (rate) in the OPD fell from 18 (0.0012%) during the 12 months before intervention III to 11 (0.0007%) from June 2008 to May 2009, with a relative reduction of 43% (Table 2); subsequent reduction was to only 3 (0.0001%) errors in 2014 (Table 1).

### Reduction of patient specimen ID errors by intervention IV

Three years after successful implementation of the barcode patient specimen ID system in the ED, a similar system was implemented on the wards in September 2010. The data collection period was divided in two periods of 12 months: period I, before implementation of the barcode patient specimen ID system (September 2009 to August 2010); and period II, after system implementation (September 2010 to August 2011). The total number (rate) of ID errors in the IPD decreased from 185 (0.0164%) to 127 (0.0112%) after intervention IV, with a relative reduction of 32% and a significant difference in the proportion of errors for the two periods (P <0.001; Table 2).

## Discussion

The study described here is a comprehensive report of hospital-wide introduction of interventions to reduce errors in patient specimen identification by the clinical laboratory of the largest medical facility in Taiwan. The main strengths of our study are as follows: a large volume of samples (over 2 million specimens handled per year); multiple service practice sites, including the OPD, ED, and IPD, covering nearly the entire hospital; and data coverage for over 10 years. Erroneous patient ID throughout the entire hospital were 511/1000000 (Table 1) in 2005, which is lower than the data reported by the College of American Pathologists (CAP) in the Q-Probes analysis of 147 clinical laboratories [12]. After these interventions, the ID errors were reduced to 15/1000000 (Table 1), which is lower than other published data [10–14, 17]. In addition, we provided solid additional support for previously reported data, affirming that rates of patient ID errors are higher for inpatients than for outpatients [10]. Most importantly, we have unequivocally demonstrated that most, if not all, patient ID errors can be prevented by implementing serial preventive interventions, namely, a restrictive specimen acceptance policy, a barcode patient identification system, and automated sample labeling combined with electronic identification systems.

Our study has clearly documented that a specimen acceptance policy that restricts relabeling of replaceable specimens is an effective intervention to improve accurate specimen identification by requiring documentation and communication between all stakeholders [24]. After this intervention, a dramatic error reduction was seen in the wards and ED (Fig 1). Weber described a similar experience and that a restrictive specimen acceptance policy is likely the most challenging and significant intervention for minimizing specimen ID errors [25]. Unfortunately, according to a CAP Q-probe survey, 42% of respondents in the United States permit relabeling of blood specimens by primary sample collection personnel [12]. It is worth pointing out that the main advantage of a restrictive specimen acceptance policy is that it requires cooperation between the clinical laboratory and nursing department.

An electronic ID system is an additional successful measure to minimize specimen ID errors [6, 8, 20, 28–33]. Reducing patient ID errors in the demanding ED environment is critical and should therefore be the first target for incorporation of an electronic ID system. One year after introduction of our barcode system to the ED specimen collection procedure, the total number of errors decreased by 78%. Using electronic processes, the potential for human error can be all but eliminated. The successful experience of using a barcode patient ID system in the ED should be repeated in the wards as well as in the ICU.

Our study is the first to report the use of an automated sample labeling system in combination with national health insurance cards, an effective method for preventing ID errors. It is noteworthy that there were still three ID errors identified from OPD in 2014. The error was occurred at the OPD phlebotomy station where specimens were labeled manually. An automated labeling system can simplify the sample collection workflow so health care personnel can be more attentive to patients, thus improving patient satisfaction. Because the national health insurance card records the patient’s ID, use of the cards can almost completely eliminate ID errors. Other errors, such as patient requisition errors in clinics, can be also checked by this system. Additional benefits include reduction of incorrect specimen containers and unlabeled specimens. Because of the great impact on patient safety in the pre-analytical phase of testing, automated labeling systems should be expanded to phlebotomy stations in the OPD and ED.

Similar to other reports [17, 20], the three most common types of patient ID errors, as described in the Material and Methods section, occurred in the current study. Most of the specimen mismatch or unlabeled specimens are easily detected during laboratory receiving or accessioning and can be corrected before analysis. In contrast, WBIT errors are usually undetectable unless discrepancies between requisitions and test results are noticed by physicians or clinical laboratory staff. Inconsistent results can also be detected by the computer-based delta check autoverification system in the clinical laboratory [9] for certain test items like creatinine or hemoglobin. The most frequent kind of ID error in 2006 was unlabeled specimens (59%), followed by specimen/requisition mismatch (36%), and WBIT specimens (5%). After serial interventions, the first two types of ID error were dramatically reduced. In 2014, the total number of ID error was 58, with unlabeled, mismatched and WBIT specimens accounting for 19%, 17% and 64%, respectively (data not shown). A total 22% of WBIT errors in 2014 were identified in the laboratory before reporting; the remaining 78% were identified by other health care staff, with no change in patient treatment, and were corrected in revised reports. The possibility remains of underestimating the actual frequency of WBIT, thus leaving room for improvement. After these interventions, the awareness among healthcare workers were increased, the computer-assisted barcode positive ID system and automated sample labeling combined with electric ID system were used. It is reasonable to speculate that a reduction in detected ID errors would also reflect a reduction in undetected errors [28].

There are other limitations besides the undetected errors in this study. The results of each intervention in the study were retrospectively analyzed and compared based on a 1-year period. The reduction in patient ID errors may not all be attributable to these interventions. During the study period, we held several interdisciplinary conferences aimed at increasing awareness among health care workers. All medical technologists and phlebotomists are required to complete continuing education credits to maintain their licensure. Such ongoing staff training and awareness programs also contribute to the reduction of errors.

## Conclusions

Patient specimen ID errors are common but preventable. Appropriate and continued communication, training, and education about the interventional procedures in this study are critical. Although there is much to accomplish before patient specimen ID errors can be eliminated altogether, dedicated effort on the part of hospital professionals together with interdisciplinary cooperation and use of new electronic ID technologies can significantly lessen such errors. These interventions can be applied to any size of hospitals. To date, we have successfully implemented these patient specimen ID interventions at all eight Chang Gung Memorial Foundation hospitals in Taiwan. We encourage other hospitals to incorporate similar interventions to further improve overall patient safety by avoiding laboratory specimen ID errors.
